# LMS-Res-YOLO: Lightweight and Multi-Scale Cucumber Detection Model with Residual Blocks

**DOI:** 10.3390/s25237305

**Published:** 2025-12-01

**Authors:** Bo Li, Guangjin Zhong, Wei Ke

**Affiliations:** 1 Faculty of Applied Sciences, Macao Polytechnic University, Macao 999078, China; p2209429@mpu.edu.mo; 2 College of Mechatronics Engineering, Zhongshan Institute, University of Electronic Science and Technology of China, Zhongshan 528402, China; 3 School of Mechanical and Electrical Engineering, University of Electronic Science and Technology of China, Chengdu 611731, China; 202221040208@std.uestc.edu.cn

**Keywords:** cucumber detection, lightweight object detection, multi-scale feature fusion, residual blocks, deep learning, YOLO

## Abstract

Efficient cucumber detection in greenhouse environments is crucial for agricultural automation, yet challenges like background interference, target occlusion, and resource constraints of edge devices hinder existing solutions. This paper proposes LMS-Res-YOLO, a lightweight multi-scale cucumber detection model with three key innovations: (1) A plug-and-play HEU module (High-Efficiency Unit with residual blocks) that enhances multi-scale feature representation while reducing computational redundancy. (2) A DE-HEAD (Decoupled and Efficient detection HEAD) that reduces the number of model parameters, floating-point operations (FLOPs), and model size. (3) Integration of KernelWarehouse dynamic convolution (KWConv) to balance parameter efficiency and feature expression. Experimental results demonstrate that our model achieves 97.9% mAP@0.5 (0.7% improvement over benchmark model YOLOv8_n), 87.8% mAP@0.5:0.95 (2.3% improvement), and a 95.9% F1-score (0.7% improvement), while reducing FLOPs by 33.3% and parameters by 19.3%. The model shows superior performance in challenging cucumber detection scenarios, with potential applications in edge devices.

## 1. Introduction

Timely harvesting of mature cucumbers is essential to preserve their quality and maximize economic benefits, making automatic cucumber detection a core technology for intelligent harvesting robots. However, greenhouse cucumber detection faces significant challenges: cucumbers share similar colors with surrounding leaves and branches, grow in dense clusters with mutual occlusion, and exhibit shape variations across varieties and growth stages. Additionally, edge devices (e.g., embedded systems on harvesting robots) have limited computational resources, requiring models to balance accuracy and efficiency.

Traditional detection methods relying on handcrafted features (color, shape, texture) struggle in complex agricultural environments. Lighting changes easily distort color-based detection [1,2], while shallow features fail to capture the contextual information needed to distinguish occluded or small cucumbers [3]. These methods also suffer from high computational complexity and slow inference speeds, unable to meet real-time detection requirements [4]. Greenhouse cucumber detection technologies based on texture and color analysis have shown limited cost-effectiveness [5], further highlighting the need for improved solutions.

Deep learning has emerged as the mainstream paradigm for object detection, which can be categorized into two-stage detectors and one-stage detectors. Represented by R-CNN [6], two-stage object detectors encompass variants such as PV-RCNN [7], Sparse R-CNN [8], and other related models [9,10,11]. While these detectors deliver high accuracy, they demand substantial computational resources primarily due to the candidate region generation step. One-stage detectors like YOLO series address this trade-off, achieving better balance between speed and accuracy. YOLOv8_n [12], as a lightweight variant, is suitable for edge deployment but still has shortcomings in complex cucumber detection scenarios: its C2f module lacks effective multi-scale feature fusion, leading to suboptimal performance on occluded or small targets; the original detection head is redundant in parameters; and standard convolutions fail to balance parameter efficiency and feature representation. In this paper, we propose a lightweight cucumber detection model based on YOLOv8_n structure. The main innovations include:

- We propose a plug-and-play multi-branch convolutional residual module, which can be applied to the backbone, neck and detection heads of YOLO series models to improve detection accuracy and recall, especially for small object detection.
- We researched the detection model’s lightweight module design and further reduced the model’s parameters, FLOPs and size while maintaining its performance.
- We created an image dataset of greenhouse cucumbers, which is useful for studying automatic picking and yield analysis.

## 2. Related Work

The cucumber target detection task is distinct from other fruit target detection tasks. In terms of appearance, cucumbers may undergo significant changes between different varieties and growth stages. Some cucumbers are long and thin, while others are short and thick. Some cucumbers are straight, while others are curved. Diversity in appearance necessitates that object detection algorithms have great generalization performance and accurate detection capabilities so that they can identify cucumbers with different appearances. In terms of the spatial distribution of cucumbers, cucumber plants often grow very densely in complex situations where cucumbers are stacked on top of each other. This density and stacking characteristics also increase the difficulty of cucumber detection. Additionally, leaves, vines, or other objects may partially obscure cucumbers, leading to poor information integrity. This partial visibility may result in a limited amount of information that object detection algorithms can use, making it difficult to accurately locate and detect cucumbers. Therefore, the algorithm needs to be robust to such conditions and able to accurately detect targets in complex scenarios. And changes in light and shadow can affect the appearance of cucumbers, resulting in changes in the brightness of captured images, which further increases the accuracy requirements for target detection models.

Lin et al. [13] constructed an efficient detection model (EFDet), which improved the detection performance of cucumber leaves in complex backgrounds by fusing feature maps at different levels. Li et al. [14] proposed an MTC-YOLOv5n method based on the YOLOv5 [15], which can effectively detect cucumber diseases in natural scenes. The model integrates Coordinate Attention (CA) and Transformer, which can reduce the interference of invalid information in the background and has strong robustness under dense fog, fine rain, and low light. Khan et al. [16] used pre-trained models VGG-19 and VGG-M for the detection and classification of cucumber leaf diseases and then extracted the most prominent features based on local standard deviation, local entropy, and local interquartile range method. They then input these refined features into multi-class support vector machines for disease recognition, as a result, five types of cucumber leaf diseases were classified. Chen et al. [17] suggested an improved YOLOv5 model for recognizing the top of a cucumber canopy, aiming to fix the issue of low accuracy in recognizing cucumber canopy vine top images. YOLOv8 [12] is a cutting-edge model that builds upon the success of previous YOLO versions, further enhancing performance and flexibility. Yang et al. [18] proposed a model based on YOLOv8, which uses deformable convolution and coordinate attention for fast cow detection, achieving mean Average Precision (mAP) of 72.9% at 62.5 frames per second (FPS). Recently, several lightweight YOLO variants have been proposed to improve efficiency for edge deployment. For instance, Light-YOLO [19] adopts channel pruning and depthwise separable convolutions to reduce model size but does not explicitly address multi-scale feature representation, limiting its effectiveness on small or occluded targets like greenhouse cucumbers. Similarly, PMDS-YOLO [20] enhances multi-scale detection through a modified PANet structure; however, its backbone still relies on conventional convolution blocks without residual multi-branch design, resulting in higher computational redundancy and weaker contextual modeling under cluttered agricultural scenes.

Multi-scale feature representation learning plays an important role in machine vision [21,22]. Object regions at different scales contain multiple levels of information. By utilizing multiscale representations, models can consider global contextual information while focusing on local details, thus generating richer and more diverse feature representations [23]. Multi-scale representations help to improve the model’s ability to understand the shape, texture, structure, and context of an object, which are prerequisites for efficient object detection. In object detection and localization tasks, multi-scale feature representations are important for detecting targets of different sizes and scales. By performing feature extraction and detection at different scales, the model is better able to capture and synthesize the target’s different scale features, which improves detection accuracy and robustness [24,25].

Most of the current research in the object detection field, including these mentioned above, focused on relatively large models, which contain large numbers of parameters and require more floating-point operations, resulting in a high computational burden. Due to the limitation of computing power and resources, these models are not suitable for application on mobile devices and systems, necessitating the development of more lightweight cucumber target detection models. Our work focuses on investigating light-weight object detection models and improving their learning efficiency so as to reach a satisfactory balance between the model size and performance.

## 3. Methodology

### 3.1. Cucumber Dataset

**Figure 1:**
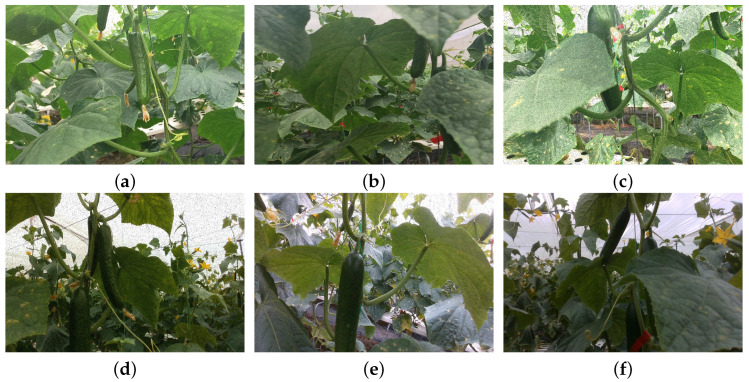
Diverse cucumber samples. (**a**) The cucumber with one side is obstructed. (**b**) The cucumber heavily shaded in the middle. (**c**) The cucumber with a large amount of missing information. (**d**) Many cucumber targets. (**e**) The cucumber located at the edge of FOV (field of view). (**f**) Cucumbers with low-level lighting.

We collected the cucumber dataset in a greenhouse farm using an Intel RealSense D435 camera. We collected images with various conditions, including angles, orientations, heights, and brightness, to enhance the model’s generalization learning ability. During this process, we also ensured the balance of various types of samples to ensure sample diversity. After removing low-quality images, a total of 2000 valid images were obtained. We Randomly divide the cucumber dataset into a training dataset, a validation dataset, and a test dataset, with a ratio of 8:1:1, and then expanded the dataset to 8000 images by applying data augmentation methods like left and right flipping, up and down flipping, Gaussian noise, and salt and pepper noise. When annotating targets in each image, we make sure that the bounding box tightly encloses the cucumber target without any missing or excessive labeling, and we ignore particularly tiny cucumber targets. Figure 1 shows the diverse cucumber samples.

### 3.2. Benchmark Model YOLOv8_n

YOLOv8 is an advanced one-stage object detector that can achieve a good balance between detection speed and precision. It supports multiple tasks, such as image classification, object detection, and image segmentation. Building upon the success of previous YOLO versions, the performance and flexibility of YOLOv8 have been greatly improved. YOLOv8 consists of YOLOv8_n, YOLOv8_s, YOLOv8_m, YOLOv8_l, and YOLOv8_x models to meet various requirements in different scenarios. YOLOv8_n has a more lightweight design compared to some other versions. It employs a simplified network structure and optimized module design to reduce the number of parameters and computations. These enable YOLOv8_n to run efficiently on mobile devices and embedded systems with limited computational resources, making it the benchmark model.

**Figure 2:**
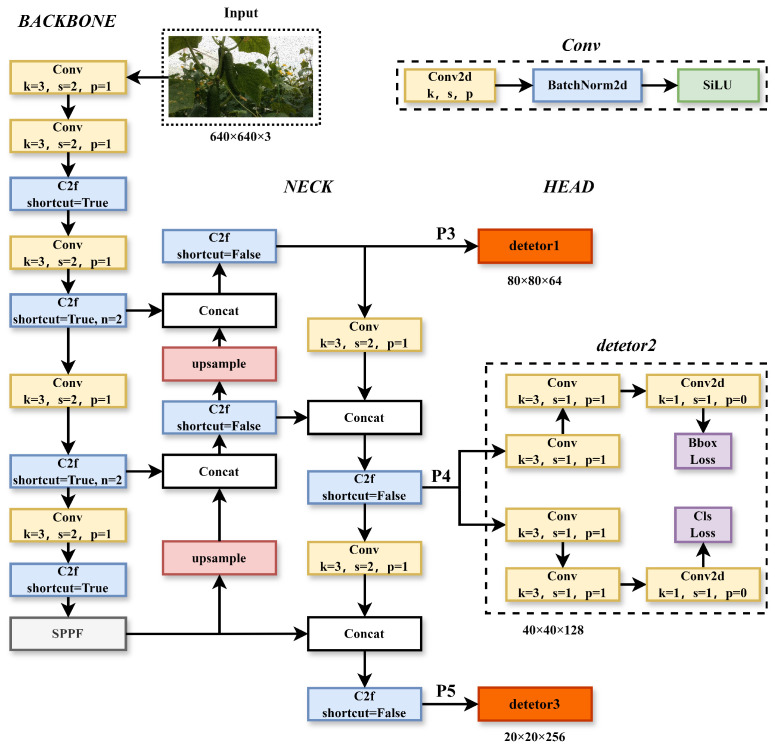
YOLOv8_n network structure. In the figure, k is the size of the convolution kernel, s is the stride, p is padding, and n is the number of repetitions of Bottleneck in C2f.

The YOLOv8_n model mainly consists of the following four parts: the backbone network, feature fusion module, detection head, and loss function, as shown in Figure 2. In addition, it also has some auxiliary elements, such as pre-processing and post-processing steps, which involve tasks like image scaling, normalization, and non-maximum suppression. YOLOv8_n uses the backbone network as its primary feature extractor to extract deep semantic features from input images. The C3 structure of YOLOv5 has been replaced with the C2f structure, which can capture more detailed gradient flow information. This change has led to a significant improvement in model performance while also ensuring that the network is lightweight. The neck part constructs a feature pyramid using the PAFPN [26] structure, enhancing feature extraction and feature representation capabilities on the backbone network’s output feature maps.

### 3.3. Our Improved Model

**Figure 3:**
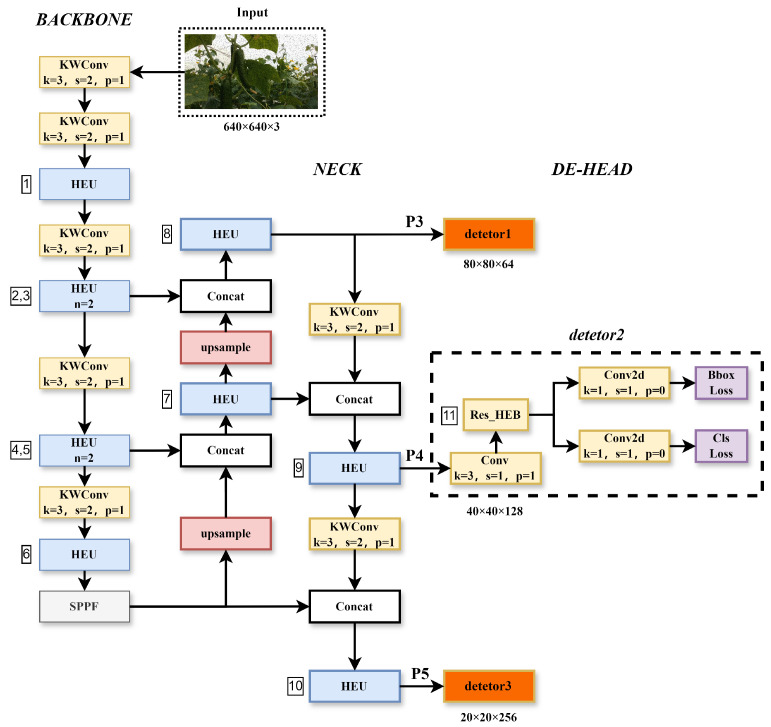
LMS-Res-YOLO network structure. In the figure, k is the size of the convolution kernel, s is the stride, p is padding, and n is the number of repetitions of Res_HEB in HEU.

In complex natural environments, YOLOv8_n performs poorly in recognizing cucumbers. Based on YOLOv8_n, the LMS-Res-YOLO model has been developed, which is a lightweight and efficient cucumber object detection model that excels at accurately identifying cucumbers in near-colored backgrounds. LMS-Res-YOLO’s multi-layered network structure captures more detail in the input data by utilizing multiple layers of feature extractors. Figure 3 illustrates the network structure of the LMS-Res-YOLO model, and the main improvements over our proposed LMS-Res-YOLO include: (1) To replace the C2f with the HEU (High-Efficiency Unit with residual blocks) to enhance the multi-scale representation ability of features, improve the nonlinear modeling ability of the network for complex problems, and reduce information loss during network transmission. (2) To replace the detection head with the DE-HEAD (Decoupled and Efficient detection HEAD with the fusion of multi-scale features) in order to further reduce the model’s size without affecting its performance. (3) To replace the Conv of the backbone and neck with the KWConv (KernelWarehouse) [27], which was proposed by Intel in 2023. It can reduce the model’s size and better balance the relationship between parameter efficiency and representation ability.

#### 3.3.1. HEU Module

**Figure 4:**
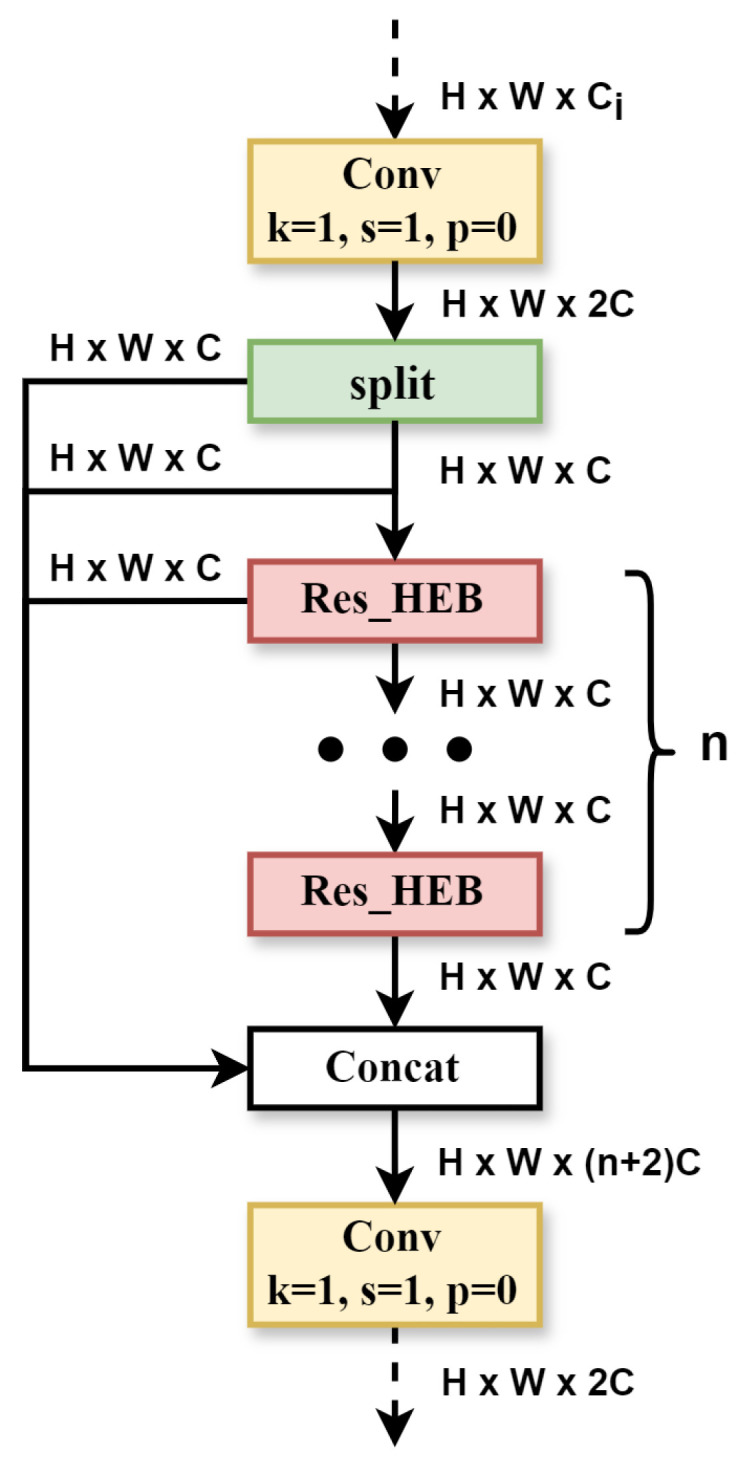
HEU module.

**Figure 5:**
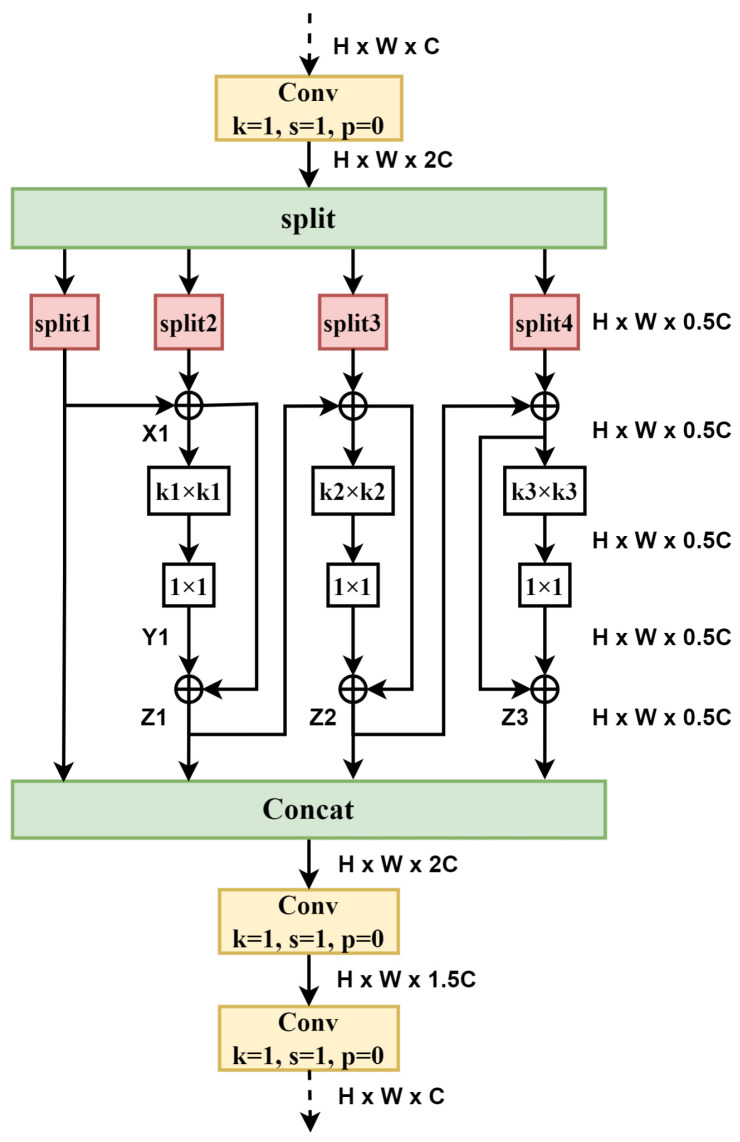
Res_HEB block.

The C2f module has some shortcomings, such as its large number of parameters and high computational complexity. The newly designed HEU is more lightweight and has further improved performance. After replacing C2f with HEU alone, there is a slight improvement in mAP@0.5, and mAP@0.5:0.95 increases by 1%, which is very commendable. The HEU structure is shown in Figure 4. It is made up of the Conv, Split, Res_HEB (High-Efficiency Block with Residual blocks), and Concat modules. In Figure 5, the Res_HEB block takes full advantage of the multi-scale representation fusion, which can make models achieve invariance to scale changes. In practice, an object’s scale may change due to factors such as distance, viewing angle, or image resolution. By extracting features at different scales, the model can better adapt to objects of different sizes, leading to increased accuracy and stability. Feature fusion techniques frequently utilize multiscale representations to obtain more global and comprehensive feature representations [28,29]. For Res_HEB, the input feature maps have dimensions of $H\times W\times C_{i}$. After the transition via a 1×1 convolution, the dimension of the output feature maps becomes H×W×2C. Next, the output is divided into four parts in the channel dimension, which are denoted as split 1, split 2, split 3, and split 4. Subsequently, split 1 and split 2 execute the element-wise tensor addition operation, creating a new tensor known as X1, which maintains the same shape. X1 then undergoes depth-wise separable convolution with a kernel size of k1 while keeping the same input and output channel numbers. This process efficiently extracts features from multi-channels, preserves spatial information, requires few parameters, and maintains the number of channels unchanged. Z1 emerges from the element-wise tensor addition between X1 and Y1. This operation belongs to residual connections [30], which is crucial to our model, and experimental results show that our model cannot converge during training without it. Then, Z1 undergoes element-wise tensor addition with split 3. Following the same process as above, Z2 and Z3 are obtained. These feature maps from split1, Z1, Z2, and Z3 are concatenated as input to two 1×1 convolutions to further obtain the cross-channel feature information.

Our LMS-Res-YOLO network uses total 13 Res_HEB blocks in the backbone, neck and detector, and the parameters of each Res_HEB are shown in Table 1, in which indexes 1 to 6 are used in the backbone and indexes 7 to 10 are used in the neck. The Res_HEB block uses a large convolutional kernel size of k1, which can capture more contextual information, share parameters over a wider receptive field, and improve the model’s generalization ability.

#### 3.3.2. DE-HEAD

The original YOLOv8 head is a type of decoupled detection head that needs separate network branches to predict the object’s category and position. This makes the model more complicated and increases the number of parameters. As shown in Figure 3, we constructed the DE-HEAD to replace the original head, reducing the head’s parameters and further making the model lightweight. Our DE-HEAD caused the learnable parameters and FLOPs (floating-point operations per second) of the model to decrease by more than 10%, while the other performance remained stable. After extracting features through the backbone network and neck network, we obtain a set of feature maps denoted as P3, P4, P5 at different scales. Then P3, P4, and P5, respectively, pass through the DE-HEAD detector. First, DE-HEAD adjusts the number of channels in the input feature maps using a 3×3 convolution. Subsequently, the adjusted feature maps are fed into a Res_HEB block, which uses the index 11 parameters in Table 1 to fuse information on different scales, thereby enhancing the representation ability of features and the model’s robustness to scale changes. Finally, feature maps go through a 1×1 convolutional layer to adjust the number of channels and then compute the loss function.

#### 3.3.3. KWConv

To further reduce the model’s FLOPs, KernelWarehouse [27], denoted as KWConv, is introduced in the positions of the backbone network and neck network. It can reduce FLOPs by 15% with constant parameter amounts. Ordinary convolutions perform poorly in terms of parameter efficiency and representation ability, while KWConv can keep a favorable balance between them. The purpose of KWConv is to significantly increase the number of kernels while reducing their dimensions. Kernel partitioning and warehouse sharing are powerful tools in KWConv. It can enhance the relationship within the same layer and across consecutive layers to improve the dependency relationship of convolutional parameters. Firstly, KWConv divided the convolution kernel into m disjoint kernel units, which have the same dimensions. Then, calculate the linear mixture of each kernel unit based on a predefined “warehouse” containing n kernel units. This warehouse is shared across multiple adjacent convolutional layers, improving parameter sharing. Finally, the corresponding m mixtures are assembled in sequence, providing higher flexibility while maintaining a lightweight design.

## 4. Experiments

### 4.1. Evaluation Metrics

We use mAP@0.5, mAP@0.5:0.95, Params, FLOPs, Precision (P), Recall (R), F1-score (F1), and Model Size as evaluation metrics in our experiments to comprehensively and objectively assess the performance of the LMS-Res-YOLO object detection model. (1) mAP@0.5 represents the average accuracy at an Intersection over Union (IoU) of 0.5 and is a commonly used evaluation metric in object detection tasks to measure the accuracy of the model in detecting targets. (2) mAP@0.5:0.95 is Similar to mAP@0.5, it takes into account a range of IoU thresholds from 0.5 to 0.95, at a step of 0.05, providing a more comprehensive evaluation of model performance by considering a series of overlap levels between predicted bounding boxes and ground truth boxes. Compared to mAP@0.5, mAP@0.5:0.95 is a more stringent evaluation metric. (3) Params refers to the number of parameters in a model, usually measured in millions (M), which represent the model’s complexity and memory requirements. In general, a larger number of parameters indicates a more complex model. (4) FLOPs represent the number of floating-point operations per inference, indicating a model’s computational workload. Lower FLOPs values often indicate lower computational complexity. (5) Precision (P) is defined as detection precision, which measures a model’s predictive accuracy, with higher values indicating greater accuracy. It is equal to the ratio of the number of correctly detected cucumber targets to the total number of detected targets in this experiment. (6) Recall (R) is defined as the ratio of the correctly detected number of cucumber targets to the ground truth number. Higher values indicate higher sensitivity. (7) F1-score (F1) is the harmonic mean of Precision and Recall, it provides a balanced measure of a model’s performance by considering both precision and recall, with a higher value indicating a better balance between the two metrics. (8) Model Size refers to the file size of the model, including model’s structure and weights.

### 4.2. Ablation Experiments

The experimental configuration is shown in Table 2.

In order to evaluate the impact of the three improvements on the overall performance of the network structure, a series of ablation experiments were conducted in this experiment. The above improvements were combined to verify reliability and effectiveness, providing guidance for model design and optimization. The results of the ablation experiments are shown in Table 3. The symbol ✓ refers to the use of the improved module. If there is no ✓ for the three improvement measures, it is the baseline model, YOLOv8_n. To ensure the objectivity of the experiment, all training processes were conducted from scratch with exactly the same hyperparameters as the baseline model YOLOv8_n.

When the three improved modules work independently, they all bring improvements to mAP@0.5 and mAP@0.5:0.95, with HEU performing the best. In terms of making the model lightweight, HEU reduced Params and FLOPs by 9% and 3.7%, respectively, but increased the model size slightly. KWconv decreased FLOPs by 14.8%, but increased Params and Model Size slightly. DE-HEAD has the most significant effect on lightweight, reducing Params, FLOPs, and Model Size by 11.3%, 14.8%, and 8%, respectively. Combining all three modules leads to improvements in all evaluation indicators, with mAP@0.5, mAP@0.5:0.95, Params, Precision, Recall, F1-score, and Model Size achieving improvement effects of 0.7%, 2.7%, 19.3%, 0.3%, 1.2%, 0.7% and 5.5%. FLOPs achieved the most significant improvement at 33.3%. The ablation experiment results demonstrate that our improvement modules can improve the original YOLOv8_n’s performance while also making it lighter-weight.

### 4.3. Comparison Experiment with Advanced Models

To further verify the effectiveness of our LMS-Res-YOLO, comparative experiments were conducted with current advanced object detection models in the YOLO series, including YOLOv5 [15], YOLOv6 [31], YOLOv7 [32], YOLOv8 [12], YOLOX [33], and YOLOv10 [34]. The experiments results are presented in Table 4 and Table 5. It is evident that in terms of accuracy, YOLOv5_s, YOLOX_m, YOLOv8_s, and YOLOv10_s achieve slightly higher performance on mAP@0.5; only YOLOv8_s and YOLOv10_s outperform LMS-Res-YOLO at mAP@0.5:0.95. However, YOLOv5_s, YOLOv8_s, and YOLOv10_s are relatively large-scale models. Compared with the proposed LMS-Res-YOLO, their Params increase by 188.5%, 358%, and 230.9%, FLOPs by 192.6%, 425.9%, and 300%, and Model Size by 143%, 280%, and 179.5%, respectively. In contrast, LMS-Res-YOLO exhibits outstanding overall performance when compared with models of similar scale (i.e., YOLOv5_n, YOLOv6_n, YOLOv8_n, and YOLOv10_n), achieving the highest accuracy in mAP@0.5, mAP@0.5:0.95, Precision, Recall, and F1-score. While YOLOv5_n has a parameter advantage (1.76 M vs. 2.43 M), our LMS-Res-YOLO demonstrates superior overall performance. Specifically, it achieves 97.9% mAP@0.5 (vs. 97.1%), a 95.9% F1-score (vs. 94.3%), and, more importantly, 87.8% mAP@0.5:0.95 (vs 80.4%)—a substantial 7.4% improvement in the more rigorous metric.

These comparative experiments confirm that LMS-Res-YOLO can effectively balance the complexity and performance of an object detection model. It requires fewer computational resources and less storage space for operation, which is critical for its application in resource-constrained devices such as mobile devices, embedded systems, and edge devices.

### 4.4. Visualization Comparison Experiments

**Figure 6:**
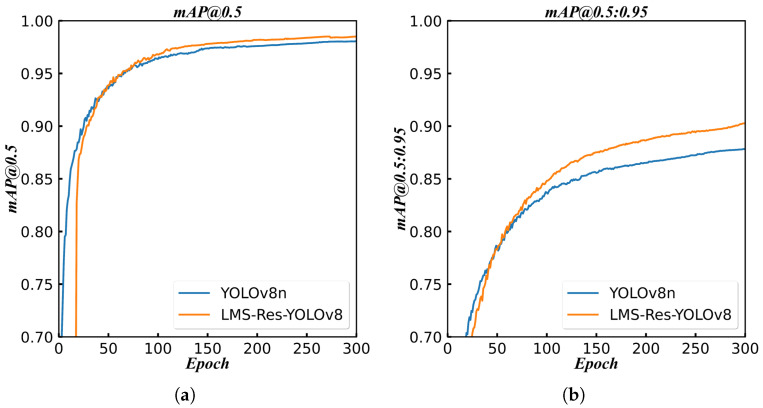
Training curve of mAP on YOLOv8_n and LMS-Res-YOLO. (**a**) Training curve of mAP@0.5. (**b**) Training curve of mAP@0.5:0.95.

**Figure 7:**
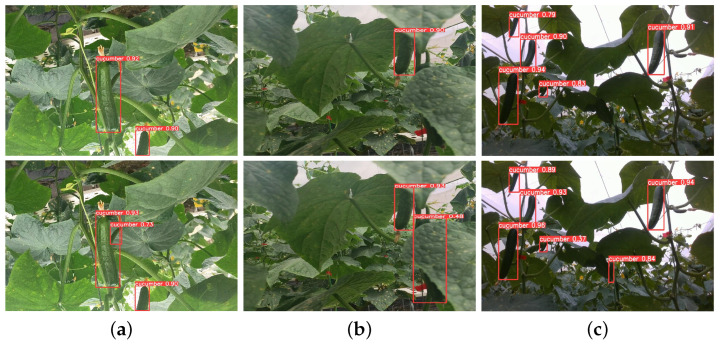
Comparison of detection results on obscured cucumbers between YOLOv8_n and LMS-Res-YOLO. The first row shows the detection results of YOLOv8_n, and the second row shows the detection results of LMS-Res-YOLO. (**a**) Overlapping cucumber. (**b**) Heavily obscured cucumber. (**c**) Obscured cucumber.

**Figure 8:**
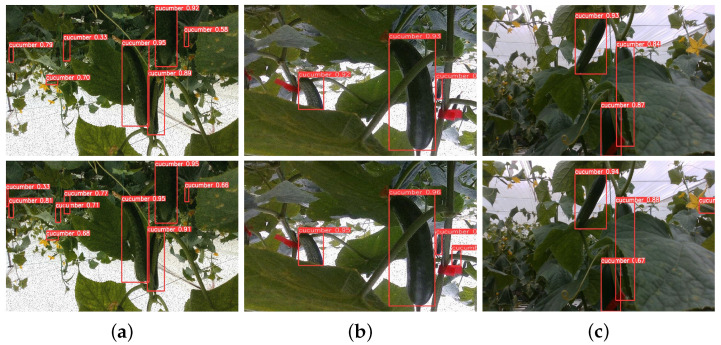
Comparison of detection results on small cucumbers between YOLOv8_n and LMS-Res-YOLO. The first row shows the detection results of YOLOv8_n, and the second row shows the detection results of LMS-Res-YOLO. (**a**) Small cucumbers at the edge of FOV and occluded small cucumbers. (**b**) Occluded small cucumbers. (**c**) Small cucumbers at the edge of FOV.

Figure 6 presents the mAP curve during training, demonstrating that after approximately 50 training epochs, the mAP results of LMS-Res-YOLO outperform those of the benchmark model YOLOv8_n. To further validate the model’s performance, additional challenging experiments were conducted between LMS-Res-YOLO and YOLOv8_n. As illustrated in Figure 7, LMS-Res-YOLO exhibits superior detection performance on occluded and overlapping cucumbers. Specifically, it can accurately detect cucumbers in scenarios such as partial overlap (e.g., Figure 7a,c) and heavy occlusion by adjacent leaves (e.g., Figure 7b). Figure 8 further highlights the advantage of LMS-Res-YOLO in small target detection: unlike YOLOv8_n, which misses some small targets, LMS-Res-YOLO achieves accurate detection of such objects, including obscured small cucumbers (e.g., Figure 8a,b) and small cucumbers growing laterally near the edge of the field of view (FOV) (e.g., Figure 8c).

Features at different scales contain semantic and detailed information at varying levels, which facilitates distinguishing cucumber targets from backgrounds or other objects. The experimental results demonstrate that our LMS-Res-YOLO exhibits stronger and more robust detection performance than YOLOv8_n. This advantage is mainly attributed to three factors: First, the HEU module’s multi-scale design enables effective feature extraction at different receptive fields, crucial for handling scale variation. Second, the Res_HEB residual blocks facilitate gradient flow, improving training stability and convergence. Third, the integration of KernelWarehouse convolution (KWConv) optimizes parameter efficiency and representation capacity through its kernel partitioning and warehouse sharing mechanism. Consequently, LMS-Res-YOLO not only improves the accuracy and comprehensiveness of cucumber detection but also enhances the ability to detect occluded targets and FOV edge targets.

## 5. Conclusions

This study intends to develop a lightweight object detection model with potential applications for cucumber detection on edge devices. First, we designed the HEU module to replace the C2f module in YOLOv8_n; this module enhances the ability of multi-scale feature representation, thereby improving detection accuracy. Second, we proposed the DE-HEAD, which effectively reduces the model’s parameters, FLOPs, and size while ensuring the stability of detection accuracy. Third, we replaced the standard convolutions in the network’s backbone and neck components with KWConv, which further enhances detection accuracy without increasing FLOPs. The experimental results indicate that the proposed LMS-Res-YOLO model outperforms the benchmark model YOLOv8_n in challenging cucumber detection scenarios. Additionally, we also demonstrated that our model has strong generalization performance on public datasets, as shown in Appendix A. Future work will focus on real-time deployment optimization on mobile terminals.

## Figures and Tables

**Table 1:** Parameters of each Res_HEB in LMS-Res-YOLO.

Index	Parameters C, [k1, k2, k3]
1	16, [7, 3, 3]
2	32, [9, 3, 3]
3	32, [9, 3, 3]
4	64, [11, 3, 3]
5	64, [11, 3, 3]
6	128, [11, 3, 3]
7	64, [11, 3, 3]
8	32, [11, 3, 3]
9	64, [11, 3, 3]
10	128, [11, 3, 3]
11	64, [7, 3, 3]

C represents the channel numbers. [k1, k2, k3] represent the kernel sizes of the three depth-wise separable convolution.

**Table 2:** Experiment hardware and software system.

Experimental Environment	Configuration
CPU	AMD Ryzen 95900X@3.70 GHz
GPU	NVIDIA GeForce RTX3090 (24 GB)
Memory	64 GB
Operating system	Windows10 64 bit
Python version	Python 3.8
Pytorch version	Pytorch1.8.0 (torchvision0.9.0)
CUDA/CUDNN	11.1/18.0.5

**Table 3:** Ablation experiments results.

HEU	KWConv	DE-HEAD	mAP@0.5	mAP@0.5:0.95	Params (M)	FLOPs (G)	P	R	F1	Model Size (MB)
			0.972	0.855	3.01	8.1	0.966	0.938	0.952	5.98
✓			0.975	0.865	2.74	7.8	0.962	0.946	0.954	6.03
	✓		0.975	0.859	3.02	6.9	0.968	0.940	0.954	6.04
		✓	0.974	0.863	2.67	6.9	0.958	0.944	0.951	**5.5**
✓	✓		**0.980**	0.874	2.76	6.6	0.968	**0.953**	0.960	6.12
✓		✓	**0.980**	0.877	**2.41**	6.6	**0.972**	0.951	**0.961**	5.55
	✓	✓	0.974	0.866	2.69	5.6	0.962	0.938	0.950	5.57
✓	✓	✓	0.979	**0.878**	2.43	**5.4**	0.969	0.949	0.959	5.65

**Table 4:** Experimental comparison results with relatively large models.

Detection Model	mAP@0.5	mAP@0.5:0.95	Params (M)	FLOPs (G)	P	R	F1	Model Size (MB)
YOLOv5_s	**0.983**	0.858	7.01	15.80	**0.970**	**0.971**	**0.970**	13.76
YOLOv6_s	0.975	0.861	18.50	45.17	0.969	0.947	0.958	38.74
YOLOv6_m	0.973	0.864	34.80	85.62	**0.970**	0.928	0.948	72.50
YOLOv7_tiny	0.978	0.843	6.01	13.20	**0.970**	0.958	0.964	11.71
YOLOX_tiny	0.951	0.732	5.03	15.23	0.935	0.914	0.924	38.70
YOLOX_s	0.977	0.847	8.94	26.76	0.954	0.942	0.948	68.51
YOLOX_m	0.980	0.873	25.28	73.73	0.963	0.953	0.958	193.35
YOLOv8_s	0.981	**0.889**	11.13	28.40	0.967	0.962	0.964	21.50
YOLOv10_s	0.981	0.879	8.04	21.6	0.966	0.957	0.961	15.79
LMS-Res-YOLO(our)	0.979	0.878	**2.43**	**5.4**	0.969	0.949	0.959	**5.65**

**Table 5:** Experimental comparison results with similar scale models.

Detection Model	mAP@0.5	mAP@0.5:0.95	Params (M)	FLOPs (G)	P	R	F1	Model Size (MB)
YOLOv5_n	0.971	0.804	**1.76**	**4.20**	0.951	0.936	0.943	**3.72**
YOLOv6_n	0.970	0.842	4.63	11.34	0.966	0.940	0.953	9.95
YOLOv8_n	0.972	0.855	3.01	8.1	0.966	0.938	0.952	5.98
YOLOv10_n	0.974	0.843	2.28	6.7	0.966	0.926	0.946	5.51
LMS-Res-YOLO(our)	**0.979**	**0.878**	2.43	5.4	**0.969**	**0.949**	**0.959**	5.65

## Data Availability

The datasets used and analyzed during the current study are available from the corresponding author upon request.

## Abbreviations

The following abbreviations are used in this paper:

YOLO	You Only Look Once
PAFPN	Path Aggregation Feature Pyramid Network
HEU	High-Efficiency Unit
Res_HEB	High Efficiency Block with Residual blocks
DE-HEAD	Decoupled and Efficient detection HEAD
KWConv	KernelWarehouse convolution
FLOPs	Floating-point operations per inference
mAP	mean Average Precision
FOV	field of view

## Appendix A

To evaluate the generalization performance of our detection model, we conducted experiments on the public datasets include PASCAL VOC2007&2012 [35], MS-COCO2017 [36] and VisDrone2019 [37]. It is worth emphasizing that MS-COCO2017 and VisDrone2019 are characterized by a large proportion of small-scale objects, which poses significant challenges to the detection ability of most existing models. All models involved in the experiments were trained from scratch to ensure the fairness and reliability of the comparison, and subsequent testing was performed under consistent evaluation settings. The experimental results from Table A1, Table A2 and Table A3 prove that our model has more advantages in the evalutation metic of mAP, especially in the more stringent mAP@0.5:0.95 compared to benchmark models with similar model sizes. Notably, this superiority is more pronounced in the small-target-rich scenarios of MS-COCO2017 and VisDrone2019, fully verifying the effectiveness of our model in addressing small object detection challenges.

**Table A1:** Comparison experiment results on VOC2007&2012 dataset.

Detection Model	mAP@0.5	mAP@0.5:0.95	Params (M)	FLOPs (G)	P	R	F1
YOLOv8_n	**0.807**	0.604	3.01	8.1	**0.819**	0.718	0.765
YOLOv10_n	0.805	0.607	**2.30**	6.7	0.815	0.723	**0.767**
LMS-Res-YOLO(our)	0.802	**0.616**	2.43	**5.4**	0.800	**0.726**	0.761

**Table A2:** Comparison experiment results on MS-COCO2017 dataset.

Detection Model	mAP@0.5	mAP@0.5:0.95	Params (M)	FLOPs (G)	P	R	F1
YOLOv8_n	0.517	0.369	3.01	8.7	0.631	0.476	0.543
YOLOv10_n	0.536	0.388	**2.30**	6.7	0.643	**0.492**	0.558
LMS-Res-YOLO(our)	**0.540**	**0.394**	2.43	**5.4**	**0.652**	**0.492**	**0.563**
**Detection Model**	**mAP@0.5:0.95 (Small)**		**mAP@0.5:0.95 (Medium)**		**mAP@0.5:0.95 (Large)**		
YOLOv8_n	0.182		0.405		0.527		
YOLOv10_n	0.184		0.424		0.558		
LMS-Res-YOLO(our)	**0.195**		**0.434**		**0.570**		

**Table A3:** Comparison experiment results on VisDrone2019 dataset.

Detection Model	mAP@0.5	mAP@0.5:0.95	Params (M)	FLOPs (G)	P	R	F1
YOLOv8_n	0.341	0.195	3.01	8.1	0.455	**0.343**	0.391
YOLOv10_n	0.341	0.199	**2.30**	6.7	0.477	0.333	0.396
LMS-Res-YOLO(our)	**0.343**	**0.200**	2.43	**5.4**	**0.479**	0.339	**0.399**
